# A high serum creatine kinase (CK)-MB-to-total-CK ratio in patients with pancreatic cancer: a novel application of a traditional marker in predicting malignancy of pancreatic masses?

**DOI:** 10.1186/s12957-023-02903-3

**Published:** 2023-01-18

**Authors:** Cong Chen, Xianchao Lin, Ronggui Lin, Heguang Huang, Fengchun Lu

**Affiliations:** Department of General Surgery, Fujian Medical University Union Hospital, Fuzhou, 350001 Fujian China

**Keywords:** Benign pancreatic tumors, Chronic pancreatitis, CK-MB-to-total-CK ratio, Creatine kinase, Pancreatic adenocarcinoma

## Abstract

**Background:**

The finding that some benign pancreatic masses mimic the imaging appearance of carcinomas poses a challenge for pancreatic surgeons. Preoperative markers that assist in the diagnosis are critical under this circumstance. Abnormal serum creatine kinase (CK) isozyme levels were reported in cancer patients, and this study aimed to explore the potential value of the CK-MB-to-total-CK ratio (CK ratio) in differentiating pancreatic cancer (PC) from benign masses when combined with carbohydrate antigen 19-9 (CA19-9).

**Methods:**

A total of 190 patients primarily diagnosed with pancreatic masses were retrospectively reviewed and assigned to the PC group and the benign pancreatic mass (BPM) group. Sixty-eight controls were enrolled for comparison. Levels of preoperative parameters, including total serum CK, CK-MB, absolute neutrophil count, absolute lymphocyte count, albumin, and CA19-9, were recorded as well as pathological information. A logistic regression model was established to assess the application value of the combination of CA19-9 and the CK ratio in diagnosis. Receiver operating characteristic (ROC) curves were constructed to evaluate the diagnostic value of the markers.

**Results:**

The CK ratio was significantly elevated in the PC group compared with the BPM group (*P* < 0.001). In the multivariate analysis, a CK ratio greater than 0.220 was a statistically significant variable for predicting malignancy of pancreatic masses (*P*=0.001). Patients with stage III/IV PC had a higher CK ratio than those with stage I/II PC (*P*<0.01). Combined detection of CA19-9 and the CK ratio produced an increased Youden index (0.739 vs. 0.815) with improved sensitivity (82.2% vs. 89.8%).

**Conclusions:**

The CK ratio is elevated in patients with pancreatic adenocarcinoma and is an independent factor predicting pancreatic adenocarcinoma. The CK ratio augments the diagnostic capacity of CA19-9 in detecting malignancy.

## Introduction

With the increased utilization of abdominal imaging and ongoing advances, the identification of pancreatic masses with or without symptoms has occurred more frequently [1, 2]. For an individual with a pancreatic mass, resection of the lesion, which may lead to lifelong morbidity and poor quality of life, should be supported by valid evidence. However, surgeons sometimes cannot draw a conclusion for a primary judgment according to clinical features and imaging characteristics [3]. Some benign masses mimic the imaging appearance of carcinoma [4, 5], such as chronic pancreatitis presenting as a focal mass, solid pseudopapillary tumor, and even special serous cystadenoma, which might represent a diagnostic dilemma with pancreatic ductal adenocarcinoma (PDAC) [6]. Notably, 5–10% of patients with suspected pancreatic adenocarcinoma (pancreatic mass) based on imaging findings who undergo resection are finally diagnosed with benign disease [7]. When the imaging approaches reach their limitations, markers that can assist in predicting malignancy are critical to obtain a better integrated assessment of pancreatic mass lesions.

Carbohydrate antigen 19-9 (CA19-9) is an indicator of aberrant glycosylation and is the best validated biomarker for pancreatic cancer (PC), with a sensitivity of approximately 80% [8]. Due to abnormal synthesis of CA19-9 in other organs, increased serum CA19-9 levels are also observed in individuals with liver cancer, cirrhosis, lung fibrosis, diabetic nephropathy, and other diseases [9]. In addition, CA19-9 is present at undetectable or at low levels in Lewis antigen-negative individuals [10]. Considering these findings, the use of CA19-9 levels to differentiate PC from benign masses is not ideal. The diagnostic value may be further improved by performing a combined measurement of CA19-9 levels and other markers.

A previous study reported that 3.2% of 846 patients with various diseases who had a creatine kinase (CK)-MB-to-total-CK ratio (CK ratio) greater than 1.0 had an established diagnosis of PC [11]. Based on the premise that low total serum CK activity appears in patients with other malignancies [12], we hypothesized that a high CK ratio is a potential marker to differentiate PC from other pancreatic masses. Very few studies have elucidated the mechanism of lower CK activity in the serum of cancer patients, and it remains unclear. Creatine kinase B-type (CKB) was reported to be overexpressed in PC tissue in a mouse model and is involved in the invasion and metastasis of PDAC [13]. However, the change in serum CK activity is not necessarily consistent with CK overexpression in the tumor tissue; for example, elevated CKB levels in tumor cytosol and low total serum CK activity were reported in breast cancer patients [14, 15]. Since a comparative study measuring serum CK levels between patients with PC and benign pancreatic lesions or normal controls is unavailable, the effect of elevated CKB levels on the serum CK level requires further investigation. Hence, this retrospective study was performed to evaluate the serum CK level in patients with PC, and we sought to discover whether combining the CK ratio and CA19-9 level would further improve the diagnostic capacity.

To the best of our knowledge, this study is the first to evaluate the utilization of the serum CK level in the preoperative differentiation between benign and malignant pancreatic masses before invasive modalities are applied.

## Methods

### Patients

**Fig. 1:**
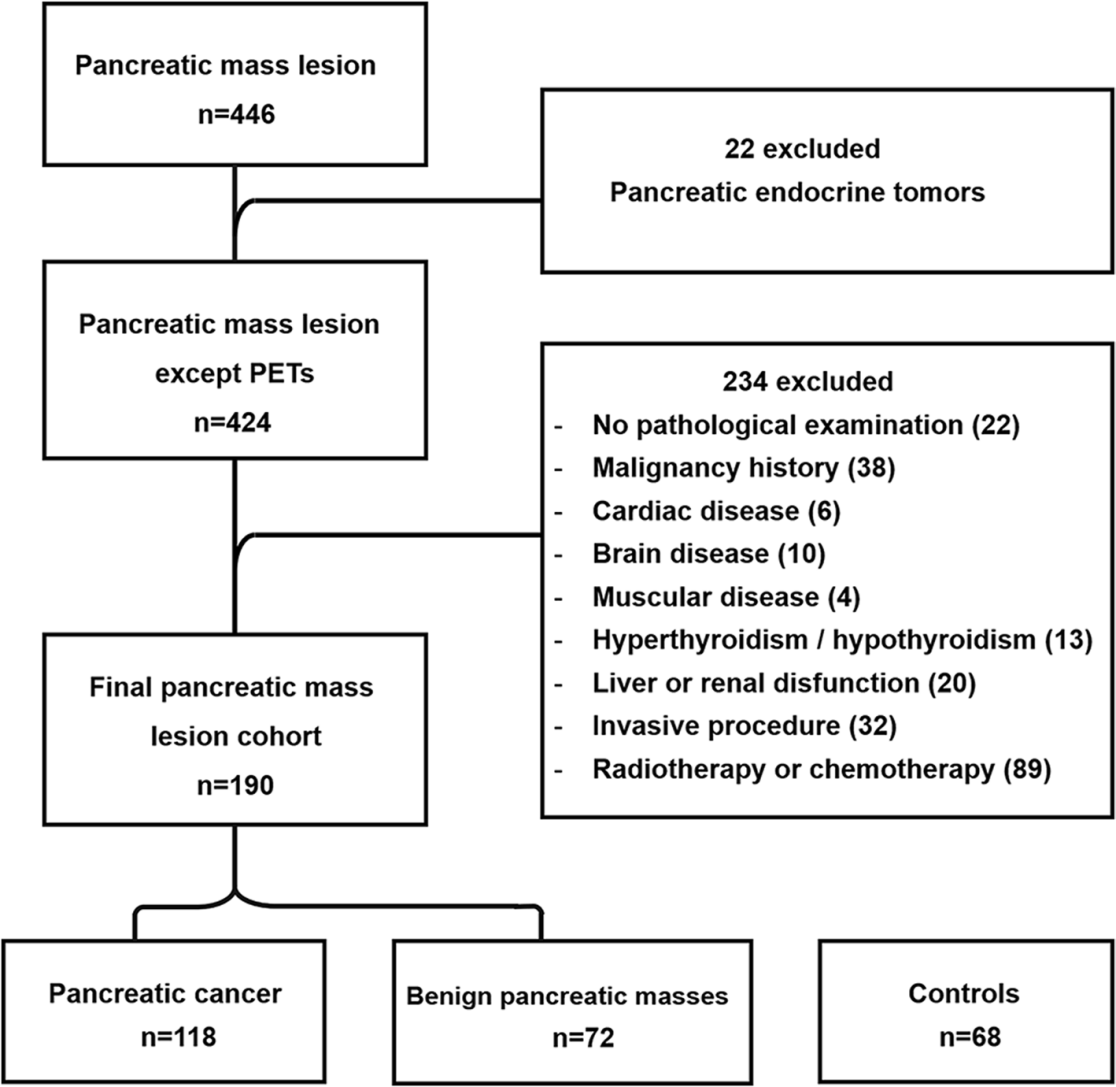
Flowchart showing the selection of the cohort for the study. PETs, pancreatic endocrine tumors

The study was approved by the Ethics Committee of Fujian Medical University Union Hospital. The records from 446 patients who were primarily diagnosed with pancreatic masses between January 2017 and November 2022 in Fujian Medical University Union Hospital were retrospectively reviewed. Patients with pancreatic endocrine tumors were excluded because they could not be simply defined as benign or malignant [16], and functional pancreatic endocrine tumors may influence creatine metabolism. The nature of pancreatic masses was finally confirmed by a biopsy of the resected specimen or a laparoscopic-assisted puncture biopsy, and patients without valid pathological confirmation were also excluded. The following exclusion criteria were applied to ensure cohort homogeneity in the preoperative values of serum total CK, CK-MB, and CA19-9: history of malignancy; cardiovascular disease: coronary artery disease and atrial fibrillation; brain disease: stroke and craniocerebral trauma; muscular disease: progressive muscular dystrophy, polymyositis, and dermatomyositis; hyperthyroidism or hypothyroidism; and liver or renal dysfunction. The application of the exclusion criteria was based on the connection of the variation in CK levels with a wide range of systemic diseases [17, 18]. In addition, all subjects who underwent an invasive procedure (for example, endoscopic retrograde cholangiopancreatography) or received radiotherapy or chemotherapy for pancreatic lesions before admission were excluded (Fig. 1). Finally, 190 patients with pancreatic masses were enrolled. In addition, 68 healthy controls who underwent a physical examination (including an abdominal computed tomography (CT) scan or ultrasound examination) during the same period were enrolled according to the same mentioned exclusion criteria, and the possibility of pancreatic diseases was excluded by the modalities and previous history.

### Data collection

Demographic data, pathological information, diabetes mellitus status, and laboratory test results (CK, CK-MB, albumin, absolute neutrophil count, absolute lymphocyte count, and CA19-9) were collected for all patients diagnosed with a pancreatic mass (missing values were estimated using the median value of the corresponding group, including 1 CA19-9 value in the PC group and 3 CA19-9 values in the benign pancreatic mass group). The same information was collected and recorded for the control group, except for pathological data and tumor marker levels. Blood samples were obtained before the operation. Blood was collected for albumin, CK, and CK-MB testing in the morning after subjects had fasted, and the levels of these parameters were measured using a Roche Cobas c 702 analyzer (Roche Diagnostics GmbH, Mannheim, Germany). CA19-9 levels were detected using a Cobas 6000 analyzer (Roche Diagnostics, Basel, Switzerland), and an automated hematology analyzer XN series (Sysmex Corporation, Japan) was used to measure routine blood cell counts. All this information was retrospectively obtained from the hospital electronic records.

### Definitions

The CK ratio is calculated as the ratio of the serum CK-MB level divided by the serum total CK level, and the neutrophil-to-lymphocyte ratio (NLR) is the ratio of the total neutrophil count divided by the total lymphocyte count. The prognostic nutritional index (PNI) was calculated as the serum albumin level (g/L) + 0.005 × total lymphocyte count (per mm^3^) [19]. Receiver operating characteristic (ROC) curves were plotted and the Youden index was used to determine the optimal cutoff values for a high CK ratio, NLR, and PNI. The cutoff values for a high CK ratio, NLR, and PNI were 0.220, 2.778, and 50.305, respectively. To compare the diagnostic value of serum total CK with the CK ratio, the lower limit of total CK was calculated, which was 62.5 IU/L. According to the manufacturer’s instructions, CA19-9 levels greater than 37 U/mL are considered abnormal.

### Statistical analysis

Continuous variables are presented as medians and interquartile ranges, as no variable was normally distributed consistently in the groups, while categorical variables are presented as whole numbers and percentages. Variations in continuous variables between two independent groups were determined using the Mann–Whitney *U* test, and the Kruskal–Wallis *H* test was used to compare three independent groups. Pearson’s *χ*^2^ test was used to compare the intergroup differences in categorical variables. Only variables with *P<*0.1 in univariate analyses were subjected to multivariate analyses for which logistic regression models were used. A logistic regression model was also established for the ROC curve to evaluate the value of the combination of the CA19-9 level and the CK ratio. Two-tailed *P* values are reported, and a *P* value <0.05 was considered statistically significant. SPSS software (version 25) was used for all data analyses.

## Results

### Demographics

One hundred and eighteen patients with pancreatic adenocarcinoma (PC group), 72 patients with a benign pancreatic mass (BPM group), and 68 controls were included in the present study. The overall patient characteristics are summarized in Table 1. The median age of patients in the PC group was significantly higher than that of patients in the BPM group (61 vs. 50 years, *P*<0.001). A significantly higher percentage of patients in the PC group had diabetes mellitus (DM) than patients in the other two groups. The CK ratio, NLR, and CA19-9 level were significantly higher in the PC group than in the BPM group, and patients in the PC group were significantly more likely to have lower CK levels, albumin (ALB) levels, and PNI values. The pathological type of PC consisted of adenocarcinoma only, and the numbers of patients with stage I to IV tumors were 17 (14.4%), 26 (22.0%), 24 (20.4%), and 51 (43.2%), respectively. The BPM group consisted of 17 patients with serous cystic neoplasms (SCNs) (23.6%), 7 with mucinous cystic neoplasms (MCNs) (9.7%), 11 with intraductal papillary mucinous neoplasms (IPMNs) (15.3%), 12 with solid pseudopapillary neoplasms (SPNs) (16.7%), 15 with mass-forming chronic pancreatitis (MFCP) (20.8%), 2 with an intrapancreatic accessory spleen, 2 with solitary fibrous tumors, 1 with a pancreatic schwannoma, 1 with hyperplasia of adipose tissue, and 4 with a pancreatic retention cyst or pseudocyst.

**Table 1:** Characteristics of patients with pancreatic cancer, benign pancreatic masses, and controls

Characteristics	PC	BPM	***P***_**1**_	Controls	***P***_**2**_^**a**^	***P***_**3**_^**b**^
	***n***=118	***n***=72		***n***=68		
Age (years)	61 (55, 67)	50 (38,58)	**<0.001**	63 (54, 70)	1.000	**<0.001**
Sex, male	79 (66.9)	39 (54.2)	0.078	46 (67.6)	0.992	0.103
DM	38 (32.2)	11 (15.3)	**0.010**	6 (8.8)	**<0.001**	0.243
ALB (g/L)	42.50 (38.30, 45.73)	44.80 (43.20, 46.83)	**0.001**	44.10 (42.70, 45.78)	**0.034**	0.223
CK (IU/L)	55.00 (44.00, 74.00)	74.50 (59.25, 99.00)	**<0.001**	98.5 (74.5, 127)	**<0.001**	**0.004**
CK-MB (IU/L)	14.95 (11.80, 18.73)	13.60 (10.83, 16.05)	>0.050	15 (12, 17.53)	>0.050	>0.050
CK ratio	0.260 (0.182, 0.366)	0.173 (0.132, 0.230)	**<0.001**	0.148 (0.109, 0.197)	**<0.001**	0.134
NLR	2.707 (1.855, 3.451)	1.779 (1.450, 2.612)	**<0.001**	1.654 (1.121, 2.171)	**<0.001**	0.114
PNI	51.55 (44.54, 54.70)	54.40 (51.65, 56.79)	**<0.001**	53.775 (51.475, 56.05)	**0.002**	1.000
Ca19-9 (U/mL)	206.10 (53.68, 638.20)	13.39 (7.82, 20.94)	**<0.001**	-	-	-
Tumor site			0.248			
Head and neck	61 (51.7)	31 (43.1)	-	-	-	-
Body and tail	57 (48.3)	41 (56.9)	**-**	-	-	-

Notes: ^a^PC vs. control; ^b^BPM vs. control

*Abbreviations*: *PC*, pancreatic cancer; *BPM*, benign pancreatic mass; *DM*, diabetes mellitus; *ALB*, albumin; *CK*, creatine kinase; *CK ratio*, CK-MB-to-total-CK ratio; *NLR*, neutrophil-to-lymphocyte ratio; *PNI*, prognostic nutrition index

Bold values are significant (<0.05)

### Multivariate analyses of factors predicting the malignancy of pancreatic masses

In multivariate analyses, a high CK ratio was an independent marker of pancreatic malignancy (OR=5.426, 95% CI: 1.960–15.023, *P*=0.001) (Table 2). In addition, an age >54 years, CA19-9 level > 37 U/mL, and high NLR were shown to be independent predictors of pancreatic malignancy, while diabetes mellitus, a low serum total CK level, and a low PNI were not associated with pancreatic malignancy.

**Table 2:** Multivariate analyses of factors predicting the malignancy of pancreatic masses

Variable	OR	95% CI	***P***
Age, >54 years	4.878	1.783–13.341	**0.002**
Sex, male	-	-	0.260
DM	-	-	0.712
CK, <62.5	-	-	0.391
CK ratio, >0.220	5.426	1.960–15.023	**0.001**
NLR, >2.778	3.863	1.294–11.530	**0.015**
CA19-9, >37	48.452	15.898–147.669	**<0.001**
PNI, <50.305	-	-	0.143

Bold values signify *P* < 0.05

### Comparison of CK ratios between different subgroups and utility of the CK ratio in association with the CA19-9 level in predicting pancreatic carcinoma

**Fig. 2:**
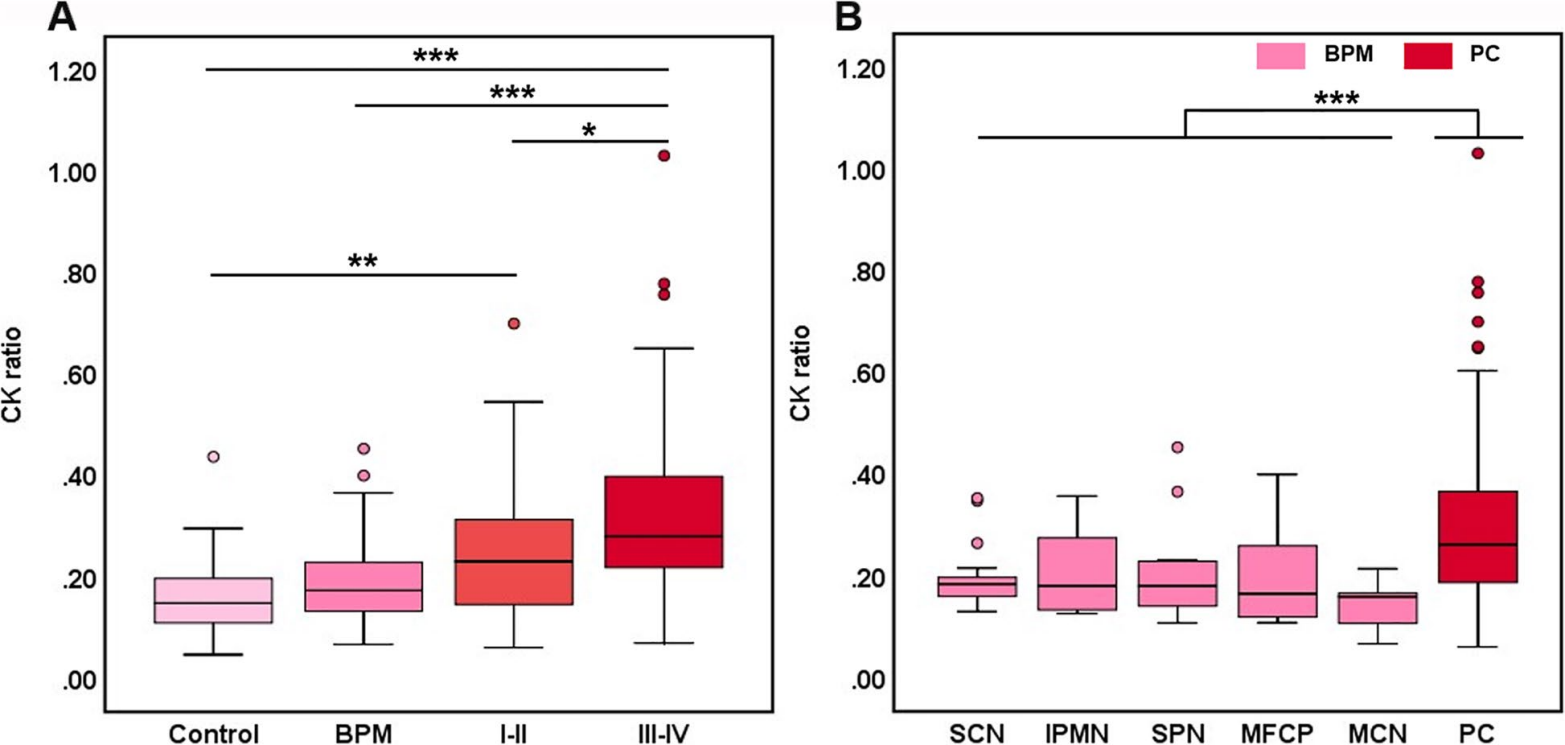
Comparisons of CK ratios among the different groups. **A** A subgroup analysis of the PC group was performed after stratification according to tumor stage (I/II and III/IV) to compare the data with the control and BPM groups (different color saturations represent the different health conditions of the subjects in the groups). The CK ratio in the III/IV group, but not the I/II group, was significantly higher than that in the BPM group. **B** The whole BPM group showed a significantly lower CK ratio than the PC group. *P<0.05, **P<0.01, and ***P<0.001. BPM, pancreatic benign mass; PC, pancreatic cancer; SCN, serous cystic neoplasm; MCN, mucinous cystic neoplasm; IPMN, intraductal papillary mucinous neoplasm; SPN, solid pseudopapillary neoplasm; MFCP, mass-forming chronic pancreatitis

The box plot in Fig. 2 compares CK ratios among the control group, BPM group, and PC groups of patients with stage I/II (I/II group) and III/IV tumors (III/IV group). Compared with the control group, the two PC groups had elevated CK ratios (*P*<0.01). Patients in the BPM group had a CK ratio (0.173, 0.132–0.230) that was significantly lower than that in the III/IV group (0.280, 0.212–0.400) (*P*<0.001). The CK ratio in the I/II group (0.231, 0.143–0.323) was higher than that in the BPM group, but the difference was not statistically significant (*P*=0.249). The difference in the CK ratio between the I/II group and the III/IV group was significant (*P*=0.025), and the latter group had higher levels than the former. The CK ratios of subgroups of the BPM group are shown in Fig. 2B, and the whole BPM group showed a significantly lower CK ratio level than the PC group (*P*<0.001).

**Fig. 3:**
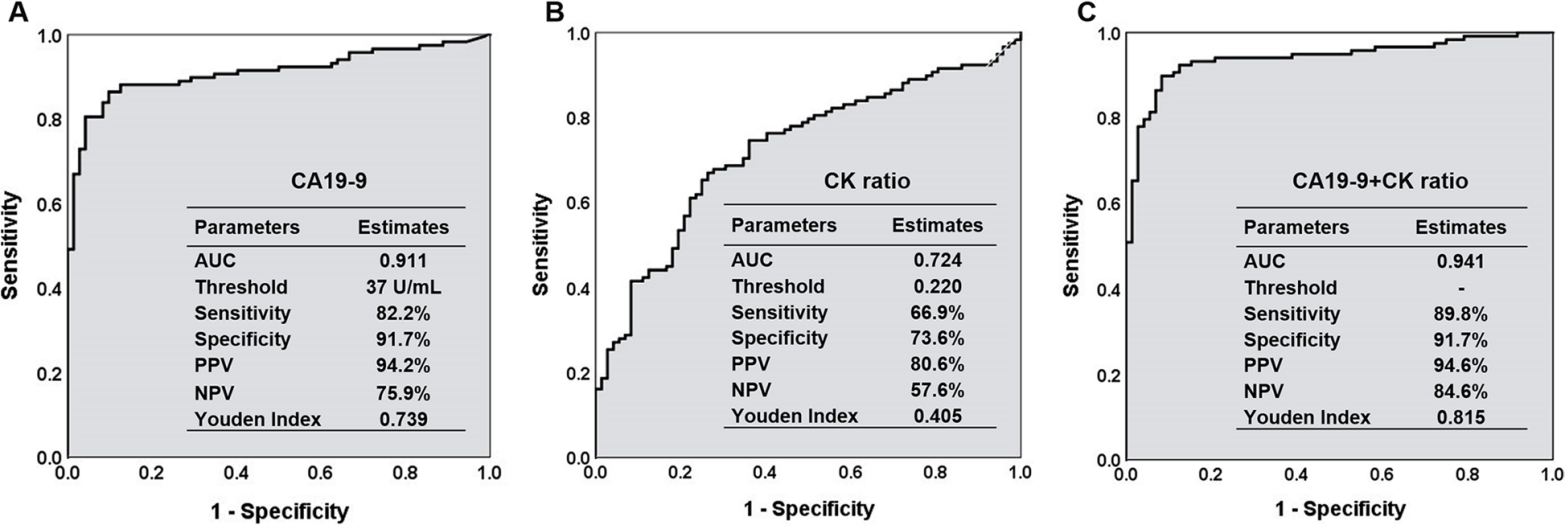
Diagnostic assessment of the CA19-9 level, CK ratio, and their combination. The combined detection of the CA19-9 and CK ratio showed an increased Youden index of 0.815 with a sensitivity of 89.8%. AUC, area under the receiver operating characteristic curve; PPV, positive predictive value; NPV, negative predictive value

The diagnostic values of the CK ratio, CA19-9, and CA19-9 combined with the CK ratio are shown in Fig. 3. The sensitivity and specificity of the CK ratio alone in predicting malignancy were 66.9% and 73.6%, respectively, and the area under the ROC curve (AUC) was 0.724. Compared with CA19-9 alone, the Youden index of the combination of the two markers improved from 0.739 to 0.815, and the sensitivity increased from 82.2 to 89.8%, whereas the specificity was consistent (91.7%). The negative predictive value (NPV) was increased from 75.9 to 84.6%, with a minimal improvement in the positive predictive value (PPV) (94.2% vs. 94.6%).

### Univariate and multivariate analyses of factors affecting the CK ratio

The data from the three groups were evaluated using a univariate analysis to determine the factors that affected the CK ratio (Table 3). The PC group was significantly associated with a higher CK ratio than the control group (*P*<0.001). The other three factors that correlated with the CK ratio value were diabetes mellitus (*P*=0.020), the NLR (*P*<0.001), and PNI (*P*=0.026). Age, sex, and BPM lesions were not significantly associated with the CK ratio. In the multivariate analysis, the NLR was an independent predictor of the CK ratio (OR=2.150, 95% CI: 1.141–4.049, *P*=0.018), but not the PNI (*P*=0.405). PC was an independent predictive factor for the CK ratio (*P*<0.001), with an OR of 7.183 (95% CI: 3.349–15.403). In the PC group, patients with stage III/IV tumors were more likely to have a high CK ratio (*P*=0.019).

**Table 3:** Univariate and multivariate analyses of factors affecting the CK ratio

Variable, ***n*** (%)	Low CK ratio	High CK ratio	Univariate analysis	Multivariate analysis		
	***n***=148	***n***=110	***P***	OR	95% CI	***P***
Age, >54 years	91 (61.5)	74 (67.3)	0.338			
Sex, male	96 (64.9)	68 (61.8)	0.615			
DM	24 (16.2)	31 (28.2)	**0.020**	-	-	0.563
Disease			**<0.001**			
PC	39 (26.4)	79 (71.8)	**<0.001**^a^	7.183	3.349–15.403	**<0.001**
BPM	53 (35.8)	19 (17.3)	0.213^b^	-	-	0.281
NLR, >2.7785	26 (17.6)	51 (46.4)	**<0.001**	2.150	1.141–4.049	**0.018**
PNI, <50.305	35 (23.6)	40 (36.4)	**0.026**	-	-	0.405
Site^1^			0.303			
Head and neck	41 (44.6)	51 (52.0)				
Body and tail	51 (55.4)	47 (48.0)				
Stage^2^			**0.019**			
I/II	20 (51.3)	23 (29.1)				
III/IV	19 (48.7)	56 (70.9)				

Notes: ^1^Data were analyzed in the PC group and BPM group. ^2^Data were analyzed in the PC group. ^a^Comparison with the control group. ^b^Comparison with the control group

Bold values signify *P* < 0.05

## Discussion

Our results revealed that total serum CK levels were significantly lower and CK ratios were significantly higher in PC patients than in patients with benign pancreatic lesions and controls. Moreover, a CK ratio >0.220 was an independent predictor of the malignancy of pancreatic masses (OR=5.426, *P*=0.001). In our study, the independent application of CA19-9 levels to detect PC showed a sensitivity of 82.2%. Considering that PC must be identified as early as possible and the price for misdiagnosing PC as a benign mass is costly, studies aiming to improve both the diagnostic sensitivity and NPV are valuable. Our study indicated that the combination of the CA19-9 and the CK ratio produced an improved sensitivity (82.2% vs. 89.8%) and a better NPV (75.9% vs. 84.6%). Moreover, a significant difference in CK ratios was observed between patients with stage I/II PC and those with stage III/IV PC, indicating that dynamic follow-up of the CK ratio in PC patients may be used to monitor tumor progression. Moving one step further, we attempted to identify whether the CK ratio can be used to detect early PC. Compared with the BPM group, the median CK ratio of the early PC (I/II stage) group was higher but was not statistically significant (*P*=0.090), indicating that its utilization in detecting early PC derived from benign tumors is limited.

Focal chronic pancreatitis in the head of the pancreas is difficult to identify intraoperatively and is one of the greatest challenges that surgical pathologists may face [20]. Many studies have tried to solve this issue through various approaches, such as specific MR techniques [21], optical spectroscopy for pathological examinations [22], and the identification of volatile organic compounds in the bile [23], and have obtained promising results. Compared with these tools, the combination of the CK ratio and CA19-9 has the advantage of being widely accessible, noninvasive, time-saving, and economic, making it suitable for the primary assessment of ill-defined pancreatic mass lesions before invasive methods are employed. Through a comparison of the rate of CK ratio > 0.220 between MFCP and PC, the CK ratio showed a promising distinguishing value (*P*=0.041, data not shown in the results). The NLR was reported to be a tool for predicting malignancy in mucin-producing pancreatic cystic neoplasms and IPMNs [24, 25] and is a common parameter used in the clinic. Our results revealed that the CK ratio was significantly lower in patients with four types of benign pancreatic neoplasms than those with PC, and ROC curves revealed that the CK ratio had a higher AUC value (0.724, 95% CI: 0.652–0.797, *P*<0.001) than the NLR (0.703, 95% CI: 0.627–0.779, *P*<0.001) (data not shown in the results).

Regarding the significant difference in the CK ratios of the respective groups (median 0.260 vs. 0.173 vs. 0.148, *P*<0.05), we considered age, sex distribution, and DM proportion as underlying confounding factors. The age gap between the PC group and the BPM group (median, 61 vs. 50 years, *P*<0.05) was possibly due to the different onset ages of the two groups [1, 26], and our analysis indicated that the age difference was not related to a low or high CK ratio (*P*=0.338). Previous reports also documented that the total serum CK level did not significantly change with age [27–29]. Additionally, sex (*P*=0.615) and DM (*P*=0.563) were not independent factors affecting the CK ratio in our analyses.

A previous study suggested that a CK ratio greater than 1.0 is mainly due to the falsely high CK-MB value [11]. Only 1 patient had a CK ratio greater than 1.0 in our study. As the total CK level rather than the CK-MB level was significantly different among the groups, the variation in the CK ratio was most likely due to the difference in the total CK level. A decreased serum total CK level appears to result from muscle wasting in cancer patients. PC is associated with the highest prevalence rate of cancer cachexia, with the highest average weight loss of approximately 15% recorded among patients with several types of cancer [30]. As the core feature of cancer cachexia, sarcopenia results from a gradual loss of muscle quantity. According to the study by Weber et al., muscle wasting in cancer patients does not appear to involve myocellular membrane damage or myolysis since the serum myoglobin and CK levels are not out of the normal range; in contrast, the values of cancer patients are decreased compared with those in healthy controls [31], similar to our result. This study also suggested that CK levels are associated with the maximal quadriceps muscle cross-sectional area with a correlation coefficient *r* of 0.55 (*P*<0.001). Analogously, a low CK level was reported to be significantly associated with a lower skeletal muscle area at the level of the 12th thoracic vertebrae [32]. The evidence indicates that a reduction in skeletal muscle mass in cancer patients is accompanied by a decrease in serum CK levels, suggesting that the CK level might be a surrogate marker for skeletal muscle mass.

Sarcopenia of PC is deduced by complex multifactorial pathophysiologic changes, and systematic inflammation is one of the most important factors [33], which might provide a physiological explanation for the low serum CK level in cancer patients. In the systematic inflammatory milieu triggered by tumors, TNF-α-induced activation of NF-κB suppresses the expression of the myogenic differentiation factor D (MyoD) mRNA at the posttranscriptional level. A binding site for MyoD to regulate has been identified in the CK-MM promoter [34, 35], and the suppression of MyoD synthesis might lead to the loss of CK-MM activity in muscle tissue. Moreover, CK-MM accounts for over 95% of total serum CK levels under normal conditions [11] and is mainly derived from skeletal muscle [36]. The pathological decrease in total serum CK levels is most likely related to changes in CK-MM and skeletal muscle.

Since the NLR might provide evidence of systematic inflammation in cancer patients [37, 38], it was analyzed in our study, and interesting results were obtained: a high NLR was significantly related to a high CK ratio (*P*<0.001) (Table 3), and the NLR was an independent factor affecting the CK ratio in the multivariate analysis. This evidence suggests that our hypothesis that malignant lesions influence skeletal muscle through inflammatory effects is reasonable. PNI was reported to be a gauge of the nutrition of cancer patients and a novel prognostic factor for PC patients [19]. In our study, we calculated the PNI and attempted to assess the relationship between the nutritional status and CK ratio. The PNI was significantly associated with a high CK ratio in the univariate analysis but was not an independent factor in the multivariate analysis (*P*=0.405).

In the clinical setting, several comorbidities of patients with pancreatic masses may perturb the CK ratio, including the well-recognized conditions of cardiovascular disease and muscular disease. In those circumstances, physicians should consider the data the CK ratio provides. For example, since glucose [39] and lipid metabolism correlate closely with both PC tumorigenesis and cardiovascular health, a sizable proportion of cancer patients also experience heart disease [40]. For patients with a high CK ratio, it is necessary to determine whether the high CK ratio is primarily caused by an abnormally high CK-MB level resulting from myocardial damage or due to a low total CK related to tumors as mentioned above. Additionally, other modalities with higher specificity should be used if necessary. For further investigation of the CK ratio, the prognostic value of serum total CK levels in lung cancer [41], breast cancer [15], and esophageal cancer [42] has been reported, and an evaluation of whether the CK ratio serves as a prognostic marker and of its performance compared with total serum CK levels would be interesting. A prognostic study was conducted in our institution to further investigate the utility of this marker.

The present study had several limitations. Due to its retrospective nature, missing variables and selection bias were possible. Only patients with definitive pathological diagnoses were enrolled in our study, and the lack of data for those patients without pathological evidence led to nonideal representativeness. However, by analyzing only pathologically confirmed pancreatic masses, we were able to definitely diagnose ambiguous masses and whether early malignancy progressed from benign lesions. Furthermore, because of its retrospective nature, only serum total CK and CK-MB levels instead of the accurate determination of serum CK isozymes were available, preventing us from confirming which isozyme contributes to the intergroup difference in CK levels. Further prospective trials should be conducted to overcome these limitations and confirm the results.

## Conclusions

As shown in the present study, the CK ratio is elevated in patients with pancreatic adenocarcinoma and is an independent predictive factor of pancreatic adenocarcinoma in patients with ill-defined pancreatic masses. Combined detection of the CK ratio and CA19-9 level augments the diagnostic capacity of the CA19-9. Nevertheless, the CK ratio must be carefully used as a supportive marker in the clinical setting because it is inevitably affected by several diseases and provides false indications. The optimal cutoffs for the CK ratio should also be tested in an independent dataset.

## Data Availability

The datasets used and/or analyzed during the current study are available from the corresponding author upon reasonable request.

## Abbreviations

CK: Creatine kinase
CK ratio: Creatine kinase-MB-to-total-CK ratio
PC: Pancreatic cancer
BPM: Benign pancreatic mass
ROC: Receiver operating characteristic
PDAC: Pancreatic ductal adenocarcinoma
CA19-9: Carbohydrate antigen 19-9
CKB: Creatine kinase B-type
NLR: Neutrophil-to-lymphocyte ratio
PNI: Prognostic nutritional index
DM: Diabetes mellitus
ALB: Albumin
SCN: Serous cystic neoplasm
MCN: Mucinous cystic neoplasm
IPMN: Intraductal papillary mucinous neoplasm
SPN: Solid pseudopapillary neoplasm
MFCP: Mass-forming chronic pancreatitis
NPV: Negative predictive value
PPV: Positive predictive value
MyoD: Myogenic differentiation factor D
